# Clinical indicators for recommending continued care to patients with neck pain in chiropractic practice: a cohort study

**DOI:** 10.1186/s12998-023-00507-y

**Published:** 2023-08-31

**Authors:** Birgitte Lawaetz Myhrvold, Nina K Vøllestad, Pernille Irgens, Hilde Stendal Robinson, Iben Axén

**Affiliations:** 1 Department of Interdisciplinary Health Sciences, Institute of Health and Society, University of Oslo, P.O. Box 1089, 0317 Blindern, Oslo Norway; 2 Et Liv I Bevegelse (ELiB), The Norwegian Chiropractic Research Foundation, Oslo, Norway; 3 Unit of Intervention and Implementation Research for Worker Health, Institute of Environmental Medicine, Karolinska Institutet, Nobels väg 13, 171 77 Stockholm, Sweden

**Keywords:** Neck pain, Continued care, Clinical indicators, Neck pain duration

## Abstract

**Background:**

Chiropractors’ clinical indicators for recommending preventive continued care to patients with low back pain include previous pain episodes, a history of long pain duration and improvement after initial treatment. Our objectives were, in a cohort of patients with neck pain, to examine whether these clinical indicators were associated with being recommended continued care beyond 4 weeks, and if so whether this recommendation was dependent of chiropractor characteristics, as well as if the number of clinical indicators influenced this recommendation.

**Methods:**

In this multi-center observational study, 172 patients seeking care for a new episode of neck pain in chiropractic practice in Norway were included between September 2015 and May 2016. The chiropractors treated their patients as per usual, and for this study, baseline data and 4-week follow-up data were used. Patient data included the clinical indicators (1) previous episodes of neck pain, (2) a history of long duration neck pain and (3) improvement four weeks after initial treatment. The recruiting chiropractors were asked at 4-week follow-up if each patient was recommended continued care, defined as care planned beyond the first 4 weeks. Univariate and multivariable logistic regression models investigated the association between clinical indicators and the continued care recommendation, as well as the influence of chiropractor characteristics on this recommendation. Cross tabulations investigated the relationship between the number of indicators present and recommendation of continued care.

**Results:**

Long duration of neck pain was the strongest clinical indicator for being recommended continued care 4 weeks after the initial treatment. Chiropractor characteristics were not associated with this recommendation. In patients with all three clinical indicators present, 39% were recommended continued care. When two and one indicators were present, the percentages of those recommended continued care were 25% and 10%, respectively.

**Conclusion:**

Chiropractors recommended continued care for patients experiencing neck pain based on their history of long pain duration, and this was not influenced by characteristics of the chiropractor. This differs from previous studies of indicators for maintenance care in patients with low back pain.

**Supplementary Information:**

The online version contains supplementary material available at 10.1186/s12998-023-00507-y.

## Background

Neck pain is a common health problem [1] and mostly characterized by recurrent episodic or persistent fluctuating pain rather than a single isolated episode [2–5]. Studies indicate that these pain patterns are associated with increased disability and reduced quality of life compared to having a single pain episode [2, 5, 6]. Thus, neck pain is also a substantial socioeconomic burden [7, 8].

To date, the prognosis of neck pain is poorly understood, and no cure exists. For patients that experience recurrent and persistent neck pain that negatively affects daily living, pain management may be the most effective approach to reduce the number and impact of relapses and maintain good function and quality of life. In chiropractic practice, patients with these pain patterns may be recommended maintenance care (MC) [9, 10]. MC is a traditional chiropractic approach described as continued care after optimal benefit is achieved in an initial care plan [11–14]. Such an initial care plan typically lasts between 2 and 4 weeks [15, 16]. The purpose of MC is to reduce the risk of relapse and maintain good daily function [11–14], the frequency is typically treatments every 3 months, and the treatment entails manual therapy and advice on lifestyle and exercise [13, 17]. Receiving MC (i.e. continued care as decided by the chiropractor) compared to receiving symptom-guided therapy (i.e. further care decided by the patient when they perceive a need) have been examined in patients with recurrent and persistent low back pain [18]. It was concluded that MC was more effective in reducing the total number of days with bothersome pain over a year’s time [18]. However, MC is currently being investigated and not included in clinical practice guidelines as a recommended evidence-based treatment strategy for prevention.

Focus groups and surveys have systematically explored chiropractors’ indications for using MC in patients with low back pain [13]. According to these studies, MC is offered to patients who have experienced *previous pain episodes*, *long pain duration*, and have shown *improvement* after initial treatment [19–21]. These indicators for MC were confirmed in an observational study of patients with low back pain and *previous pain episodes* was found to be a strong predictor for recommending MC [22]. In addition, the recommendation of MC may also depend on the chiropractor and the clinical setting. Chiropractors who were trained in the US compared to Europe, as well as chiropractors with more experience and clinic ownership, tend to recommend MC more frequently and to a greater extent to their patients [19].

Most studies on clinical indications and efficacy of MC have been performed in patients with low back pain [13]. One study explored the effectiveness of preventive spinal manipulative care with and without a home exercise program, in comparison to no treatment in patients with neck pain [23]. However, the study did not use the clinical indicators as criteria for receiving MC. Musculoskeletal pain, including low back and neck pain, share many common features on clinical course, prognostic factors, and prognosis [2, 24–29]. Hence, there are reasons to believe that patients with neck pain are managed similarly to patients with low back pain [30, 31], and this study aimed to investigate if the clinical indicators found for patients with low back pain apply to patients with neck pain in chiropractic practice. We used data from a cohort study of patients with neck pain. After four weeks following inclusion, chiropractors assessed if further treatment was recommended or not [15, 16]. This recommendation was used as a proxy for MC.

Specifically, the objectives were (1) to investigate whether the clinical indicators identified for low back pain were associated with being recommended continued care for patients with neck pain, (2) whether this recommendation was dependent on chiropractor characteristics, and (3) to examine if the number of identified clinical indicators influenced this recommendation.

## Method

### Design and setting

This study was based on data from a one-year longitudinal observational multicenter cohort study designed to identify clinical course patterns and prognostic factors in patients with neck pain consulting chiropractors in Norway. The Norwegian Regional Committees for Medical and Health Research Ethics (2015/89) approved the study. The study was reported according to the STROBE statement [32].

### Recruitment

Chiropractors across Norway were recruited through The Norwegian Chiropractors’ Association’s newsletters, general assembly, and social media groups, there were no specific criteria for the clinic or chiropractor. Those who signed up for the study were given written material and a brief workshop regarding the primary study objectives, inclusion, and exclusion criteria, as well as procedures. A total of 66 chiropractors from 43 clinics in all four Norwegian health regions were recruited, representing urban and rural areas, and each of them recruited 1–11 consecutive patients with neck pain. This broad approach ensured a diverse sample of Norwegian chiropractors and their neck pain patients. Between September 2015 and May 2016, patients who were aged 18 years and older, consulting the chiropractor for the first time with an episode of neck pain with or without arm pain and were fluent in Norwegian, were eligible for participation. We excluded patients with a suspicion of serious pathology (i.e., malignancies, inflammatory diseases, fractures, or nerve root involvement that required referral to surgery). For this study, we used data collected with digital questionnaires at baseline and at 4-week follow-up. The treatment was according to the chiropractor’s choice and included various methods where manipulation and soft tissue techniques were most frequently used. However, this study did not address treatment, and it was up to the chiropractor to determine the appropriate treatment plan. Although there was no blinding of patients or chiropractors involved in the study, the questionnaires were completed independently and without the presence of researchers. More information on the recruitment process, procedures, and the baseline questionnaire (Additional file 1) used for the cohort has been previously published [5, 6, 33–36].

### Measurements

#### Patient-reported data

The baseline questionnaire included sociodemographic data such as gender (female/male) and age (years), pain history and symptom variables (pain intensity, disability, and number of musculoskeletal (MSK) pain-sites). Pain intensity was rated on the numeric rating scale (NRS) [37]. The Neck Disability Index (NDI) assessed disability [38]. MSK pain-sites were assessed by the Nordic Pain Questionnaire (NPQ) counting the number of musculoskeletal pain sites [39]. Furthermore, at baseline two of the identified clinical indicators (previous neck pain episodes and duration of current neck pain) were collected. Previous neck pain episodes included four response options: ‘no, first time’, ‘yes, 1–3 times previously’, ‘yes, more than 3 times previously’ or ‘yes, more or less chronic neck pain’. The four responses were dichotomized into ≤ 3 episodes (0) and > 3 episodes the previous year [1]. History of neck pain duration was assessed by the Örebro Questionnaire and included 10 response options: ‘0–1 weeks’, ‘1–2’, ‘3–4’, ‘4–5’, ‘6–8’ and ‘9–11 weeks’ and ‘3–6 months’, ‘6–9 months’, ‘9–12 months’ or ‘more than one year’ [40]. The response options were dichotomized into < 30 days (0) and ≥ 30 days [1].

Information regarding patient’s subjective improvement has, in previous studies been collected during the fourth consultation following the initial treatment [18, 22]. In the present study, the participants reported improvement at four weeks used as a proxy for improvement at the fourth consultation. During this initial treatment period, most patients received between 1 and 5 consultations (Additional file 2). At 4-week follow-up, we used the Global Perceived effect (GPE) to assess improvement after initial treatment [41]. It was scored by a 7-point Likert-scale ranging from ‘recovered’, ‘much improved’, ‘slightly improved’, ‘no change’, ‘slightly worse’, ‘much worse’ to ‘worse than ever’. We added ‘not sure’ as an additional option. The GPE scores were dichotomized into improved (‘recovered’ or ‘much improved’, [1]) and non-improved (‘slightly improved’, ‘no change’, ‘slightly worse’, ‘much worse’, ‘worse than ever’ or ‘not sure’, (0)). GPE is a suitable outcome measure in neck pain research, and when dichotomized, it is considered clinically relevant[42, 43]. Additionally, at 4-week follow-up, patients reported pain intensity (NRS) and disability (NDI).

#### Chiropractor-reported data

At baseline, chiropractors reported their gender (female/male), age (years), the country for their chiropractic education, and the graduation year. The following countries were used ‘United Kingdom’, ‘Denmark’, ‘USA’, and ‘Australia’. For analyzing, we treated the country of chiropractic education as a categorical variable and used ‘United Kingdom’ as reference. To determine the number of years in practice, we used the graduation year as a proxy and subtracted it from the year for data-collection (2015). We analyzed the number of years in practice as a continuous variable.

#### Outcome measure

At 4-week follow-up, the chiropractors answered if further treatment was planned for each patient. The question (called ‘continued care’) included four response options: ‘no further treatment planned’, ‘no further treatment planned but patient will contact when treatment is needed’, ‘yes further treatment planned but patient must contact for continued care’ and ‘yes, appointment(s) have been set up for continued care’. These four categories were dichotomized into ‘no continued care’ (‘no further treatment planned’, ‘no further treatment planned but agree patient will contact when treatment is needed’ or ‘yes further treatment planned but patient must contact for continued care’) and ‘yes continued care’ (‘yes, appointment(s) have been set up for continued care’).

### Data analysis

Descriptive analyses were used to characterize the study sample, the analyzed study sample and the stratified sample dichotomized by the continued care variable. We used mean values and standard deviations for continuous variables and frequencies for categorical variables. Differences in patients’ characteristics between the dichotomized continued care outcome variable were analysed by t-tests for normally distributed variables and by Chi-squared test for categorical variables.

#### Associations between clinical indicators, chiropractor characteristics and outcome

We performed the statistical analyses in three steps consisting of univariate and multivariable logistic regression models [44]: (1) we initially examined the crude association between each single indicator variable (previous neck pain episodes, duration of current neck pain or improvement at four weeks) and outcome (continued care) using univariate logistic regression analyses, (2) we then assessed the crude association between each chiropractor characteristics (gender, age, country of educational institution and number of years in practice) and outcome (continued care) using univariate logistic regression analyses, and (3) we conducted multivariable logistic regression analyses to examine the associations of both indicator variables and chiropractor characteristics with outcome (continued care) while controlling for patient age and gender. Reference of the outcome variable was ‘no continued care’.

The outcome parameter was the odds ratio (OR) with 95% confidence interval (CI). An additional outcome measure was discrimination by the Area Under the Receiver-operating Curve (AUC) (with 0.5 representing no discrimination beyond chance and 1 representing perfect discrimination) [45]. Thus, an AUC of 0.50 indicates no discrimination, values greater than 0.50 but less than 0.70 suggest poor discrimination, values between 0.70 and 0.80 indicate acceptable discrimination, values between 0.80 and 0.90 suggest excellent discrimination, and values equal to or greater than 0.90 indicate outstanding discrimination [46]. We assessed the AUC to measure to which extent the model assigns a higher probability of ‘continued care’ to a patient in contrast to a patient of ‘no continued care’. Consequently, we compared the AUC of the two models in step 2 to quantify the benefit of chiropractor characteristics to identify patient’s recommended continued care. We used the method by Riley et al. to calculate for the efficient sample size for multivariable logistic regression modelling [47]. In the present study, three candidate clinical indicators and four chiropractor characteristics were selected a priori based on previous research [22]. We pre-specified the anticipated Nagelkerke R^2^ (0.15) and used overall outcome proportion of 0.5. For logistic models, this specifies a sample size (n) of 103 for these indicators. Our total sample size included 172 patients. Thus, we anticipated that our study would provide meaningful estimates of predictive performance.

Cross-tabulations were used to investigate how the number of indicators (i.e., previous neck pain episodes, duration of current neck pain and improvement four weeks after initial treatment) related to the recommendation for continued care. This descriptive approach enabled an assessment of the association between multiple indicators and the likelihood of recommending continued care.

At the 4-week follow-up assessment, four chiropractors failed to complete the questionnaire, resulting in the absence of outcome data for eight patients. We excluded these eight patients from the cross-tabulation and regression analysis, thus our analyzed sample included 164 participants. Importantly, there were no significant differences observed between the chiropractors who completed the 4-week follow-up questionnaire and those who did not, regarding their chiropractic characteristics or the number of patients recruited into the study. Furthermore, no substantial differences were found between the analyzed sample and the excluded patients (see Additional file 3).

Clustering effect in studies happens when patients treated by the same chiropractor have similar outcomes on factors such as characteristics and treatment protocols. Since only a small number of patients were recruited by each chiropractor it deemed unnecessary to conduct multi-level analysis.

All analyses were performed in STATA, version 16 (StataCorp LP, College Station, Texas).

## Results

### Descriptive

The chiropractors (n = 66) were mainly female (64%), and the mean age was 39 (sd 8) years. Most chiropractors graduated from a chiropractic college in the United Kingdom, and the majority had worked more than 5 years in practise (Additional file 4).

The study sample included 172 patients with neck pain. After 4 weeks, 37 (23%) had an appointment set up for continued care (Table 1), and these were mainly female, reported more previous pain episodes, a longer pain duration, higher pain intensity and a higher number of MSK pain-sites compared to patients without recommendation of continued care. Moreover, at 4 weeks follow-up, patients with an appointment for continued care reported higher pain intensity and NDI scores compared to patients with no appointments for continued care. A description of all four categories of the continued care outcome variable can be seen in Additional file 5.

**Table 1:** Characteristics of the study sample and stratified by the dichotomized continued care

	Study sample	No continued care	Yes, continued care	P-value
	n = 172 (100%)	n = 127 (77%)	n = 37 (23%)	
Baseline characteristics				
Gender, n (%) female	120 (70)	84 (66)	31 (84)	0.039*
Age (years), mean (sd)	43 (13)	42.13 (12)	46 (13)	0.081
Previous episodes of neck pain, > 3 episodes (%)	105 (61)	73 (58)	29 (78)	0.024*
Duration of current neck pain, n (%) ≥ 30 days	103 (60)	61 (48)	29 (81)	0.001*
Pain intensity (0–10), mean (sd)	4.6 (2.4)	4.2 (2.4)	5.6 (1.9)	0.002*
NDI (0–50), mean (sd)	11.8 (7.8)	11.5 (8.1)	12.8 (6.6)	0.356
MSK pain-sites, (0–10), mean (sd)	4.3 (2.1)	4.1 (2.1)	5.1 (1.9)	0.009*
4-week characteristics				
Pain intensity (0–10), mean (sd)	2.6 (2.2)	2.3 (2.1)	3.7 (2.1)	0.0005*
NDI (0–50), mean (sd)	7.6 (5.8)	7.1 (6.0)	9.6 (4.8)	0.018*
Improvement four weeks after initial treatment, n (%)	109 (63)	84 (66)	21 (57)	0.295

sd (standard deviation); NDI (Neck Disability Index); MSK (musculoskeletal)

The estimates of each indicator in the univariate models and when combined in the multivariable model are shown in Tables 2 and 3, respectively. In the univariate regression analyses, long duration of neck pain showed a significant association with being recommended continued care. In the multivariable regression model, combining the three indicators, only long duration of neck pain was found to be significantly associated with continued care. This significant association persisted when we included chiropractor characteristics in the model (Table 4). However, none of the chiropractor characteristics (gender, age, country of educational institution and number of years in practice) were statistically associated with recommending continued care (Table 4, and Additional file 6). Previous neck pain episodes and improvement four weeks after initial treatment were not statistically significantly associated with the outcome.

**Table 2:** Results of the univariable logistic regression analyses between each single indicator and the outcome variable continued care (reference: no continued care)

	OR (95% C.I)
Indicators	
Previous neck pain episodes	2.18 (0.90, 5.27)
Long duration of current neck pain	**5.10 (2.00, 12.98)**
Improvement four weeks after initial treatment	0.71 (0.33, 1.53)

Significant associations are marked in bold

**Table 3:** Results of the multivariable logistic regression analysis between indicators and the outcome variable continued care (reference: no continued care)

	OR (95% C.I)
Indicators	
Previous neck pain episodes	2.11 (0.82, 5.42)
Long duration of current neck pain	**5.04 (1.96, 12.96)**
Improvement four weeks after initial treatment	1.17 (0.50, 2.74)
AUC	0.76 (0.67, 0.84)

Significant associations are marked in bold

Ref. = reference

Controlled for patient age and gender

**Table 4:** Results of multivariable logistic regression analysis between indicators, chiropractor characteristics, and the outcome variable continued care (reference: No continued care)

	OR (95% C.I)
Indicators	
Previous neck pain episodes	2.04 (0.76, 5.45)
Long duration of current neck pain	**6.59 (2.36, 18.35)**
Improvement four weeks after initial treatment	1.60 (0.62, 4.17)
Chiropractor characteristics	
Gender (Ref. female)	0.43 (0.13, 1.37)
Age (years)	1.02 (0.80, 1.30)
Country of educational institution United Kingdom Denmark USA Australia	Ref. 1.70 (0.53, 5.35) 0.64 (0.15, 2.73) 0.74 (0.06, 9.22)
Number of years in practice	1.04 (0.83, 1.32)
AUC	0.80 (0.72, 0.88)

Significant associations are marked in bold

Ref. = reference

Controlled for patient age and gender

The discrimination accuracy described by AUC was 0.76 in the multivariable logistic regression model including clinical indicators alone (Table 3). Adding chiropractor characteristics to the model, the significant association between long duration of current neck pain and the outcome persisted and the AUC of the model increased to 0.80 (Table 4). However, none of the chiropractor characteristics was found to be significantly associated with the outcome.

Approximately 25% of the analyzed study sample was recommended continued care, independent of how many indicators were present. The cross tabulation showed that when patients had all three indicators, 39% of them were recommended continued care (Table 5). The percentage decreased to 25% when two indicators were present and further dropped to 10% when only one indicator was present.

**Table 5:** The relationship between number of indicators presents and the outcome variable continued care

	No continued care	Yes, continued care	Total
Indicators			
All 3 indicators, n (%)	17 (60)	11 (39)	28 (100)
2 indicators, n (%)	61 (75)	20 (25)	81 (100)
1 indicator, n (%)	43 (90)	5 (10)	48 (100)
No indicators, n (%)	4 (100)	0	4 (100)
Missing, n (%)	2 (67)	1 (33)	3 (100)
Total, n (%)	125 (78)	36 (22)	164 (100)

Indicators: Previous neck pain episodes, long duration of current neck pain and improvement four weeks after initial treatment

Missing: incomplete information on indicators

## Discussion

This study examined if previously identified clinical indicators (previous pain episodes, a long pain duration and improvement after initial treatment) for recommending MC to patients with low back pain also applied to patients with neck pain who were recommended continued care beyond 4 weeks. Having a long duration of current neck pain was found to be the only clinical indicator to predict if continued care was recommended to the patient by the chiropractor. However, a recommendation for continued care was not associated with chiropractor characteristics. We found that when all three clinical indicators were present, 39% of patients were recommended to receive continued care. As the number of indicators decreased, the likelihood of the recommendation decreased as well, suggesting the relationship between indicator count and the recommendation for continued care.

Our findings indicate that clinical indicators for recommending continued care to patients with neck pain are different from those used for recommending MC to patients with low back pain. A study investigating the rationale for MC in a low back pain population found that patients perceive MC to prevent recurrences and help them remain as pain free as possible [17]. This matches the beliefs of chiropractors where low back pain episode frequency and duration (both over the past year and of the present episode) were considered important factors influencing the recommendation of MC [12, 19]. In a study of patients with low back pain, previous episodes were found to be the best predictor of MC recommendation [22]. The same study found that patients reporting a long duration of their low back pain were not statistically associated with such a recommendation.

A long duration of pain and previous pain episodes are likely to be highly correlated, and a history of long pain duration has been associated with an unfavorable outcome [48]. Thus, it seems appropriate that the severity and persistence of a complaint are related to an approach that involves recommending continued care. Moreover, most predictors of unfavorable outcome are similar for low back and neck pain [26, 28]. Due to similarities between neck and low back pain, the discrepancy between our results and previous findings for low back pain may be a random finding. Nevertheless, this study needs to be replicated in a new cohort to confirm the results.

Interestingly, reporting improvement with initial treatment by the fourth week was not found to influence continued care. Thus, despite that improvement with initial treatment has been suggested as an important indication for MC by chiropractors in the Nordic countries [22], it was not found to be an indicator for continued care in our study. There seems to be a variety of aspects associated with the use of MC and the evidence is conflicting regarding whether improvement with initial treatment (i.e., the patient reports benefit from initial treatment) is a necessary indicator [11, 14, 22].

The accuracy of the multivariable regression models was examined using the AUC. Overall, the information provided by the indicators allowed continued care to be distinguished from not continued care with a moderate degree of accuracy (AUC = 0.76). Thus, many patients with all three indicators were frequently regarded as not being candidate for continued care. When adding chiropractor characteristics in the model, the AUC increased to 0.80. Even acknowledging that chiropractor characteristics might impact the improved AUC outcome, no statistically significant association between chiropractors and the outcome was found. Moreover, it is important to note that the confidence intervals for the AUC values overlapped suggesting the observed increase from an initial AUC of 0.76 to 0.80 may not be statistically significant. Therefore, the practical significance of this improvement remains uncertain.

One study found that the initiation of MC is a shared decision between the patient and the chiropractor [49]. At baseline, we observed some differences in characteristics of patients with continued care planned compared to patients with no continued care planned. Previous research indicate that the intent of MC is to prevent future pain episodes, and it is logical that patients with a long pain duration and previous episodes may be recommended continued care, based on their previous pain experience [12, 17, 21]. In addition, clinicians may (perhaps even subconsciously) consider present bio-psycho-social factors to contribute to the development of persistent pain [50–52] before recommending continued care. For instance, studies have found that patients with a less favorable psychological profile report better outcomes from a MC approach [53, 54]. Current evidence suggest that clinicians assess patients’ psychological distress together with their previous pain history, initial treatment effectiveness and patient preferences when identifying patients with low back pain suitable for MC [55].

Characteristics of a chiropractor (such as their gender, age, place of training, year of graduation, number of years in practice, and specialty) will be the same exposures for patients visiting a specific chiropractor, but different from those of another chiropractor. A Danish study found that place of training and number of years in practice were associated with the use of MC [19]. However, we did not find any significant associations between chiropractor characteristics and outcome. In our study, most chiropractors had recruited three patients or less, therefore high, and low use of continued care could not be explored. Moreover, studies investigating evidence-based practice in chiropractors found that traditional knowledge and expert opinions were used to the same extent as clinical guidelines when managing patients [56–58]. We hypothesize that these aspects may well (consciously or subconsciously) affect what patients are recommended continued care, which may have had an impact on the results of this study.

### Strengths and limitations

The study was a multicenter study gathering data on patients with neck pain from many Norwegian chiropractors, thus likely to reflect clinical practice and result in good external validity. We consider our sample to be representative when compared to previously published work [59]. Our study population includes patients with neck pain commonly seen in primary care. The baseline characteristics and outcomes reported by our chiropractic patient population align with other cohort studies from primary and general care populations [2, 3, 60], indicating that our sample can be generalized to other neck pain populations.

We used digital questionnaires that required all questions to be answered, eliminating the issue of missing data. Our study had a limitation regarding loss to follow-up, as 4% of the included patients lacked information from chiropractors regarding continued care at the 4-week follow-up. This may raise a question about possible attrition bias. To address this, we tried to contact all chiropractors (by phone and/or mail) and ask about reasons for not completing the questionnaire. The primary reason reported was lack of time. There were no obvious differences observed between chiropractors caring for patients with or without information in the analyzed sample. Additionally, the excluded patients without information showed only minimal variations from the analyzed sample, indicating a limited risk for attrition bias to impact the study findings.

We tested the associations in both univariate and multivariable models. The sample size (n=164) was considered sufficient to explore the predictive performance and strength of association between three indicators, chiropractor characteristics and outcome [47]. A potential limitation to consider is the lack of statistical testing and consideration of potential confounding factors, which could have influenced the observed relationship between indicator count and the recommendation for continued care.

Another potential limitation is that the chiropractors in the study were not specifically asked whether they considered the individual patient as candidate for MC or if they recommended a preventive approach. The analysis is based on their recommendation of continued treatment. Our results are therefore not directly comparable with those of MC studies. Neck pain is a heterogeneous condition with distinct clinical features and various responses to treatment, so the intent of continued care may have been diverse and not solely preventive. For instance, patients being slow responders to initial care plans may have required extended treatment plans, and as a result, been deemed suitable for continued care. Therefore, patients may have been recommended continued care with different objectives and, not solely preventive. However, we do believe that a recommendation for continued care may serve as a proxy for a MC recommendation.

One may argue that a follow-up period of four weeks is too short to consider a continued care approach for patients with low back or neck pain. We found that patients recommended for continued care were more severely affected at baseline and at 4-week follow-up. These patients reported higher pain intensity, a longer pain duration, and a higher number of MSK pain-sites compared to patients not being recommended continued care. One may hypothesize that the chiropractors were sensitive to these hallmarks and identified these patients as a subgroup with a more severe pain affliction. Thus, continued care would be a reasonable option to manage their condition.

### Clinical implications

In patients with neck pain, a long duration of pain was a strong clinical indicator for the chiropractor to recommend continued care. Thus, the duration of neck pain may have important clinical implications.

## Conclusion

The clinical indicators for recommending continued care to patients with neck pain in chiropractic practice differ from those used when recommending MC to patients with low back pain. We found that the strongest clinical indicator for the chiropractor to recommend continued care was a long duration of neck pain. None of the included chiropractor characteristics had a significant impact on this recommendation. Only 39% of patients presenting with previous neck pain episodes, a history of long neck pain duration and improvement four weeks after initial treatment had an appointment for continued care. Our results suggests that more knowledge is needed about the possible differences in recommending continued care for patients with neck.

### Electronic supplementary material

Below is the link to the electronic supplementary material.

Supplementary Material 1

Supplementary Material 2

Supplementary Material 3

Supplementary Material 4

Supplementary Material 5

Supplementary Material 6

## Data Availability

The data that supports the findings of the current study are not publicly available due to data protection policies. Data are however available from the corresponding author on reasonable request.
