# Supplementary material for: Clinical indicators for recommending continued care to patients with neck pain in chiropractic practice: a cohort study
Source: Chiropr Man Therap. 2023 Aug 31;31:33. doi: 10.1186/s12998-023-00507-y (PMC10472687; doi:10.1186/s12998-023-00507-y)
Supplement: Supplementary file 2 — Supplementary Material 2 [file 12998_2023_507_MOESM2_ESM.docx]

Additional file 2. The reported the number of visits (n) for each patient between baseline and 4-week follow-up

| **Number of visits from baseline to 4-week follow-up** | **n (%)** |
| --- | --- |
|  |  |
| 0 | 23 (14) |
| 1-5 | 115 (70) |
| 6-10 | 24 (15) |
| >10 | 2 (1) |
| Total | 164 (100) |
