# Supplementary material for: Clinical indicators for recommending continued care to patients with neck pain in chiropractic practice: a cohort study
Source: Chiropr Man Therap. 2023 Aug 31;31:33. doi: 10.1186/s12998-023-00507-y (PMC10472687; doi:10.1186/s12998-023-00507-y)
Supplement: Supplementary file 3 — Supplementary Material 3 [file 12998_2023_507_MOESM3_ESM.docx]

Additional file 3. Characteristics of the analyzed study sample and the excluded patients

|  | Study sample | Excluded sample |
| --- | --- | --- |
|  | (n=164) | (n=8) |
| Baseline characteristics | | |
|  | | |
| Gender, n (%) female | 115 (70) | 5 (63) |
| Age (years), mean (sd) | 43 (12) | 48 (17) |
| Previous episodes of neck pain, >3 episodes (%) | 102 (61) | 3 (38) |
| Duration of current neck pain, n (%) ≥30 days | 90 (54) | 3 (38) |
| Pain intensity (0-10), mean (sd) | 4.6 (2.4) | 4.8 (2.3) |
| NDI (0-50), mean (sd) | 11.8 (7.8) | 11.0 (7.4) |
| MSK pain-sites, (0-10), mean (sd) | 4.3 (2.1) | 4.8 (2.6) |
|  |  |  |
| 4-week characteristics | | |
|  | | |
| Pain intensity (0-10), mean (sd) | 2.6 (2.2) | 3.0 (1.8) |
| NDI (0-50), mean (sd) | 7.6 (5.8) | 7.3 (5.4) |
| Improvement four weeks after initial treatment, n (%) | 105 (63) | 4 (50) |
| sd (standard deviation); NDI (Neck Disability Index); MSK (musculoskeletal) | | |
