# Supplementary material for: Clinical indicators for recommending continued care to patients with neck pain in chiropractic practice: a cohort study
Source: Chiropr Man Therap. 2023 Aug 31;31:33. doi: 10.1186/s12998-023-00507-y (PMC10472687; doi:10.1186/s12998-023-00507-y)
Supplement: Supplementary file 4 — Supplementary Material 4 [file 12998_2023_507_MOESM4_ESM.docx]

Additional file 4. Characteristics of the chiropractors

| Chiropractors (n=66) | | Chiropractors (n=4) |
| --- | --- | --- |
| Study sample | | **Lost to follow-up** |
| Baseline characteristics | |  |
| Gender, n (%) female | 39 (64) | 1 (25) |
| Age (years), mean (sd) | 38.6 (8.4) | 41.5 (13.4) |
| Country of educational institution, n (%)  United Kingdom  Denmark  USA  Australia | 36 (56)  17 (26)  9 (13)  4 (5) | -  3 (75)  -  1 (25) |
| Number of years in practice, mean (sd)  0-1 year, n (%)  2-5 years  6-10 years  11-19 years  20 years or more | 12.7 (8.7)  4 (7)  10 (16)  18 (30)  16 (26)  13 (21) | 15.5 (17.7)  -  1 (25)  1 (25)  -  2 (50) |
| Mean number of patients recruited | 2.0 (1.9) | 2 (1.7) |
| sd (standard deviation) | | |
