# Supplementary material for: Clinical indicators for recommending continued care to patients with neck pain in chiropractic practice: a cohort study
Source: Chiropr Man Therap. 2023 Aug 31;31:33. doi: 10.1186/s12998-023-00507-y (PMC10472687; doi:10.1186/s12998-023-00507-y)
Supplement: Supplementary file 5 — Supplementary Material 5 [file 12998_2023_507_MOESM5_ESM.docx]

Additional file 5. Characteristics of the analyzed study sample and stratified by the four categories of continued care

|  | Study sample | No further treatment planned | No further treatment planned but agree patient will contact when treatment is needed’ | Yes, further treatment planned but patient must contact for continued care | Yes, appointment(s) have been set up for continued care | P-value |
| --- | --- | --- | --- | --- | --- | --- |
|  | (n=164) | (n=38) 23% | (n=65) 40% | (n=24) 15% | (n=37) 23% |  |
| Baseline characteristics | | | | | | |
| Gender, n (%) female | 115 (70) | 26 (68) | 41 (63) | 17 (71) | 31 (84) | 0.180 |
| Age (*years*), mean (sd) | 43 (12) | 46 (13) | 40 (12) | 48 (13) | 46 (13) | 0.009* |
| Previous episodes of neck pain, >3 episodes (%) | 102 (63) | 22 (59) | 39 (60) | 12 (50) | 29 (78) | 0.116 |
| Duration of current neck pain, n (%) ≥30 days | 90 (56) | 14 (38) | 31 (48) | 16 (67) | 29 (81) | 0.001* |
| Pain intensity (0-10), mean (sd) | 4.6 (2.4) | 3.9 (2.2) | 4.7 (2.4) | 3.5 (2.7) | 5.6 (1.9) | 0.002* |
| NDI (0-50), mean (sd) | 11.8 (7.8) | 10.8 (8.3) | 11.7 (8.4) | 11.8 (7.6) | 12.8 (6.6) | 0.752 |
| MSK pain-sites, (0-10), mean (sd) | 4.3 (2.1) | 3.9 (2.3) | 4.3 (2.1) | 3.8 (1.9) | 5.1 (1.9) | 0.042* |
| 4-week characteristics | | | | | | |
| Pain intensity (0-10), mean (sd) | 2.6 (2.2) | 2.3 (2.2) | 2.1 (2.2) | 2.8(1.9) | 3.7 (2.1) | 0.003* |
| NDI (0-50), mean (sd) | 7.6 (5.8) | 6.9 (6.6) | 6.3 (5.4) | 9.4 (6.2) | 9.6 (4.8) | 0.001* |
| Improvement four weeks after initial treatment, n (%) | 105 (64) | 25 (66) | 47 (72) | 12 (59) | 21 (57) | 0.180 |
| sd (standard deviation); NDI (Neck Disability Index); MSK (musculoskeletal)  *Statistically difference between the four categories of continued care | | | | | | |
