# Supplementary material for: Clinical indicators for recommending continued care to patients with neck pain in chiropractic practice: a cohort study
Source: Chiropr Man Therap. 2023 Aug 31;31:33. doi: 10.1186/s12998-023-00507-y (PMC10472687; doi:10.1186/s12998-023-00507-y)
Supplement: Supplementary file 6 — Supplementary Material 6 [file 12998_2023_507_MOESM6_ESM.docx]

Additional file 6. Results of the univariable logistic regression analyses between each single chiropractor characteristics and the outcome variable continued care (reference: no continued care)

|  | OR (95% C.I) |
| --- | --- |
| Chiropractor characteristics |  |
|  |  |
| Gender (Ref. female) | 0.47 (0.12, 1.88) |
| Age (years) | 1.00 (0.93, 1.07) |
| Country of educational institution  United Kingdom  Denmark  USA  Australia | Ref.  1.39 (0.32, 6.01)  2.89 (0.32, 26.06)  0.96 (0.09, 10.47) |
| Number of years in practice | 0.95 (0.89, 1.02) |
| Ref. = reference | |
